# Extended Overview of Ocular Phenotype with Recent Advances in Hypohidrotic Ectodermal Dysplasia

**DOI:** 10.3390/children9091357

**Published:** 2022-09-06

**Authors:** Michele Callea, Stefano Bignotti, Francesco Semeraro, Francisco Cammarata-Scalisi, Jinia El-Feghaly, Antonino Morabito, Vito Romano, Colin E. Willoughby

**Affiliations:** 1 Pediatric Dentistry and Special Dental Care Unit, Meyer Children’s Hospital, 50139 Florence, Italy; 2 Department of Medical and Surgical Specialties, Radiological Sciences, and Public Health, Ophthalmology Clinic, University of Brescia, 25121 Brescia, Italy; 3 Regional Hospital of Antofagasta, Azapa, Antofagasta 5935, Chile; 4 Departments of Dermatology and Pediatrics, University of Rochester, Rochester, NY 14627, USA; 5 Department of Pediatric Surgery, Meyer Children’s Hospital, 50139 Florence, Italy; 6 Genomic Medicine, Biomedical Sciences Research Institute, Ulster University, Coleraine BT52 1SA, UK

**Keywords:** ectodermal dysplasia, hypohidrotic ectodermal dysplasia, ocular surface disease, meibomian glands, dry eye, ectodysplasin-A

## Abstract

The term ectodermal dysplasias (EDs) describes a heterogeneous group of inherited developmental disorders that affect several tissues of ectodermal origin. The most common form of EDs is hypohidrotic ectodermal dysplasia (HED), which is characterized by hypodontia, hypotrichosis, and partial or total eccrine sweat gland deficiency. HED is estimated to affect at least 1 in 17,000 people worldwide. Patients with HED have characteristic facies with periorbital hyperpigmentation, depressed nasal bridge, malar hypoplasia, and absent or sparse eyebrows and eyelashes. The common ocular features of HED include madarosis, trichiasis, and ocular chronic surface disease due to dry eye syndrome, which manifests clinically with discomfort, photophobia, and redness. Dry eye is common in HED and results from a combination of ocular surface defects: mucus abnormalities (abnormal conjunctival mucinous glands), aqueous tear deficiency (abnormalities in the lacrimal gland) and lipid deficiency (due to the partial or total absence of the meibomian glands; modified sebaceous glands with the tarsal plate). Sight-threatening complications result from ocular surface disease, including corneal ulceration and perforation with subsequent corneal scarring and neovascularization. Rare ocular features have been reported and include bilateral or unilateral congenital cataracts, bilateral glaucoma, chorioretinal atrophy and atresia of the nasolacrimal duct. Recognition of the ocular manifestations of HED is required to perform clinical surveillance, instigate supportive and preventative treatment, and manage ocular complications.

## 1. Ectodermal Dysplasia

### Definition and Classification

Ectodermal dysplasia (ED) is a term used to describe a group of heterogenous congenital disorders characterized by alteration of development in two or more structures of ectodermal origins involving at least one among hair, teeth, nails or sweat glands. The Freire-Maia classification divides Eds into two categories: group A, which includes all entities with alterations in two or more of the four cited structures, and group B, which includes those entities with alterations in only one of the structures cited in addition to a defect in another ectodermal structure [1,2].

Eds could be associated with developmental failure in other body parts of ectodermal origin, such as the thyroid gland, the mammary glands, the adrenal medulla, the anterior pituitary gland, the thymus, the central nervous system, the cornea, the conjunctiva and the meibomian glands [3,4].

ED syndromes are characterized by anomalies of ectoderm-derived structures in association with defects of other embryonic origins, such as cleft lip/palate. Pure ectodermal dysplasias show symptoms related to structures of ectodermal origin alone [5]. Although the first classifications were based on the phenotypic aspects of the disease, providing useful guidance to the clinician on the treatment of affected individuals, the evolution of the genetic and molecular knowledge unveiled how some conditions are linked at a molecular level. In 2017, an international working group met to develop a classification approach that would incorporate phenotype, inheritance, and molecular etiology, including developmental pathways or structural assembly, to organize and cluster EDs [6].

The first genetic abnormality identified as causing ED was a mutation with loss of function of the ectodysplasin (*EDA*) gene that disrupts the encoded protein EDA-A1 [7]. Subsequently, other pathogenic variants were discovered in the EDA receptor (EDAR), in the EDARADD “EDAR-associated death domain” [8] and TRAF-6 “TNF receptor-associated factor 6” [9]. More than 200 types of ED have been described so far, with the X-linked hypohidrotic ED (XLHED) being the most common form; in Denmark, a cross-sectional study reported 1224 possible cases of HED between 1995 and 2010 with 146 clinically diagnosed cases, 988 possible HED cases and 90 confirmed at a molecular level. The prevalence of molecularly confirmed XLHED cases was 1.6:100,000 [10].

A recent study by Gökdere et al. aiming to further elucidate a correlation between the genotype and phenotype of XLHED through in vitro assays concluded that the absence of circulating EDA levels is associated with a null variant in the EDA gene. On the other hand, the presence of circulating EDA does not rule out ED; this is likely explained by the fact that normal levels of a functionally impaired protein can lead to a similar ED phenotype [11].

On another note, prenatal diagnosis of ED has been discussed through multiple case reports over the past couple of decades, most recently in an article by Li et al. The authors proposed that in fetuses with a family history of ED, ultrasound assessment of the tooth germ, maxilla and mandible during the second trimester is a useful prenatal diagnostic tool [12].

Patients affected with HED are at risk for life-threatening hyperthermia. Survival into childhood and beyond is associated with severe dental abnormalities as well as chronic developmental, respiratory, cutaneous, ocular, and psychosocial disorders [13]. To date, there are no approved therapies to restore function in disorders like XLHED. In mouse and dog XLHED models, administration of a single course of an EDA-A1 replacement protein (EDI200) resulted in permanent correction of the key phenotypic features, providing the first hope for effective, targeted therapy. This new approach has prompted EDI200 studies in XLHED patients, which could lead to a novel paradigm for the rescue of the phenotype of a human developmental disorder [13].

In a promising study, the recombinant protein EDA (Fc-EDA) was administered intra-amniotically in three affected patients: in twins with a double dose at the 26th and 31st week of gestation and in a single-dose fetus at the 26th week of gestation. The twins, born prematurely at week 31, and the infant, born at week 39, demonstrated normal sweating ability and developed no XLHED-related symptoms at the 14th and 22nd months of age [14].

The ongoing EDELIFE clinical trial is an international non-randomized, multicenter clinical trial aiming to investigate the efficacy and safety of substitutive therapy administered intra-amniotically as a treatment for unborn XLHED male subjects. The protein ER-004 acts as a substitute for endogenous ectodysplasin 1 protein (EDA1). The trial is at present in Phase 2 (*NCT04980638*) with 20 estimated participants and a primary completion date expected to be in June 2023.

## 2. Hypohidrotic Ectodermal Dysplasia

### 2.1. Definition

Hypohidrotic ectodermal dysplasia (HED/EDA1; MIM#305100) is the most common form of ED. HED is characterized by hypodontia or anodontia (lack of several or all teeth), hypotrichosis (little or no hair growth on the scalp), and hypohidrosis (partial or total eccrine sweat gland deficiency) [15]. HED is a heterogeneous group of disorders and is estimated to affect at least 1 in 17,000 people worldwide [15]. The most prevalent form of HED is inherited in an X-linked recessive pattern; however, autosomal dominant and autosomal recessive forms have been described, albeit at a much lower frequency [16].

### 2.2. Genetics and Molecular Basis

HED can be caused by mutations in at least one of various genes: EDA1, encoding ectodysplasin A (EDA), EDAR, encoding for EDA receptor, EDARADD, which is the EDAR-associated death domain protein, or NEMO (NFκB essential modulator). These mutations affect components of a tumour necrosis factor (TNF)-like signalling pathway that is initiated by binding of ectodysplasin (EDA) to its transmembrane receptor (EDAR), which connects to a canonical TNF signalling cascade through a dedicated adapter protein, ultimately leading to the stimulation of NF-κB. The gene responsible for XLHED (XL-HED; MIM*305100) is named *ectodysplasin-A* (*EDA*; MIM*300451; Xq12-q13), which encodes a protein that is involved in the normal development of ectodermal appendages including hair, teeth, and sweat glands. Defects in the molecular structure of ectodysplasin-A may inhibit the action of enzymes necessary for the normal development of the ectoderm and/or its interaction with the underlying mesoderm [17].

At least eight splice variants of human *EDA1* have been identified, and the genomic structure of *EDA1* has been elucidated [17]. All *EDA1* mRNAs appear to originate from alternative splicing events that preserve exon 1. The *EDA1* cDNA was predicted to encode a 391-residue protein, of which 256 amino acids were encoded by the ‘new’ exons. The two longest transcripts, EDA-A1 and A2, differ from each other by just two amino acids that are either included or excluded, dependent on the alternative usage of splice donor sites in exon 8. The longer EDA-A1 form corresponds most closely to the Ta-A form, both being of equal length; the putative protein is 94% identical to the protein mutated in the Tabby mice (Ta-A) and includes a transmembrane domain, a collagen-like domain with 19 repeats of a Gly-X-Y motif in the presumptive extracellular domain [18,19]. EDA-A and EDA-B proteins are homologous to the Ta isoforms, Ta-A and Ta-B, respectively. The EDA-A isoforms are functionally important and critical for EDA pathology [18].

The protein family was named ectodysplasin-A [20] and is proposed to represent a new subgroup among the membrane-associated collagenous type II transmembrane proteins. Previously known members of this group include the MARCO protein, type XIII and XVII collagens, the macrophage scavenger receptor and complement component C1q [18]. Their deduced protein sequences, therefore, contain a common *n*-terminus and transmembrane domain and are likely to belong to the class II transmembrane proteins, as described for EDA-O [20] and Ta-A [7,21].

Mutations in the ectodysplasin-A receptor (*EDAR*; MIM*604095; 2q12.3), which forms a ligand-receptor pair with ectodysplasin, and EDAR-associated death domain (*EDARADD*; MIM*606603; 1q42-q43), which interacts with the death domain of EDAR and links the receptor to signalling pathways downstream, are associated with both autosomal dominant and autosomal recessive forms of HED. Mutations in *EDAR* can result in autosomal dominant HED (hypohidrotic/hair/nail type; MIM# 129490) and autosomal recessive HED (hypohidrotic/hair/tooth type; MIM#224900), whereas mutations in *EDARADD* result in autosomal recessive HED (hypohidrotic/hair/tooth type; MIM#614940) [22].

Among HEDs, there are some syndromes that may manifest with midfacial defects: mainly cleft lip, cleft palate, or both. The most recognized entities are EEC syndrome (ectodermal dysplasia, ectrodactyly and cleft lip/palate syndrome; MIM#129900), Hay–Wells or AEC syndrome (ankyloblepharon, ectodermal dysplasia and cleft lip/palate syndrome; MIM#106260) and Rapp-Hodgkin syndrome (MIM#129400), all of which are caused by heterozygous mutations in the *TP63* gene.

### 2.3. Clinical Manifestations

The main clinical features of HED are hypohidrosis (diminished sweating), hypodontia (developmental absence of one or more teeth) and hypotrichosis (sparse hair). Male patients affected with XLHED have poor thermoregulation due to reduced sweating, which ultimately leads to overheating; the latter may lead to febrile seizures and is more common during the hot season and in geographical regions with higher average temperatures [23]. The mortality rate in early childhood due to overheating is about 30% [24].

Affected individuals show hypodontia or anodontia (absence of all teeth). Diagnosis is often delayed until the teeth fail to erupt at the expected age (6–9 months) or the teeth that erupt are peg-shaped, conical, or knife-edged in shape. The mucosa of the oral cavity is prone to atrophic inflammation, and individuals may have swallowing difficulties.

The hair is fragile, thin, light-coloured and slow-growing; there may be hypotrichosis with a patchy distribution in addition to madarosis (loss of eyebrows and/or eyelashes). The skin is exfoliating, dry and thin, with generalized xerosis and/or eczematous plaques with some areas of hyperkeratosis. The periorbital and perioral skin (skin around the e4yes and the mouth) is typically wrinkled and hyperpigmented. Moreover, a higher prevalence of atopic diseases, particularly atopic dermatitis, and food allergies, was recently highlighted in an epidemiologic survey on anhidrotic ED and HED patients in Japan [25]. On the other hand, a report of a Mexican family in 2015 identified 7 members with HED with some phenotypic characteristics not previously described, including feet with wide, short and widely spaced toes, hypoplastic, convex and unpolished nails and moderate to severe plantar hyperkeratosis [26].

Some individuals affected by HED may have distinctive facies, including depressed nasal bridge (saddle nose deformity), everted nose, prominent forehead and lips, malar hypoplasia, hypertelorism (abnormally large distance between the eyes), epicanthus (skin fold of the upper eyelid) and prognathism (protrusion of the lower jaw). Abnormalities in mucous membrane function lead to frequent respiratory tract infections and changes in nasal secretions from concretions (solidified secretions in the nasal and aural passages) in early infancy to large mucous clots [27]. Common otorhinolaryngological manifestations include chronic infections such as rhinitis, pharyngitis, otitis media, as well as hearing loss, epistaxis, and dysphonia [28]. Gastroenteric gland hypoplasia in HED can result in dysphagia and constipation [27]. Patients typically have normal physical and psychomotor development. Interestingly, in a recent report on XLHED in India, global developmental delay was noted in a high proportion of patients and was associated with various EDA pathogenic variants [29].

In heterozygous female carriers of XLHED, the expression of signs and symptoms depends on the different levels of X chromosome inactivation [30]. Female carriers may have altered dentition, marked hypodontia, and sparse and patchy hair. These patients may show mosaic hypohidrosis or abnormal skin temperature due to abnormal peripheral vascular perfusion. A diagnostic test useful for the identification of heterozygous patients is the starch and iodine sweat test, where, when performed on the back of patients, a pattern of V-stripes conforming to Blaschko’s lines appears (pathways of epidermal cell migration and proliferation during the development of the fetus) [31].

## 3. Ocular Manifestations of HED

In HED, the affected ocular structures are of ectodermal origin: eyelids, nasolacrimal system, conjunctiva, corneal epithelium, lens, and lacrimal glands [32]. The ocular features range from mild involvement with limited symptoms to severe and sight-threatening lesions. The most common eye manifestations of HED include a reduction or complete absence of the eyelashes and eyebrows (madarosis), a misdirection in the growth of the eyelashes (trichiasis), recurrent inflammation of the eyelids (blepharitis), dry eye syndrome with photophobia, burning sensation and redness [4]. Sight-threatening complications result from ocular surface disease and can include corneal ulceration, perforation, and subsequent scarring [15].

### 3.1. Dry Eye Disease

The ocular surface is a complex functional system consisting of the tear film, the epithelium of the cornea and conjunctiva, the main and accessory lacrimal glands, the meibomian glands (modified sebaceous glands), the eyelashes with their associated glands of Moll and Zeis, the components of the eyelids responsible for the blink, and the nasolacrimal duct [33]. The tear film, a key component of the ocular surface, is represented by the trilaminar model, in which are described a surface lipid layer produced by the meibomian gland, a central aqueous layer produced by the main and accessory lacrimal glands and a basal mucin layer made by the conjunctival goblet cells. The meibomian glands are responsible for the secretion of most tear film lipids, which are also produced from the glands of Moll and Zeiss on the eyelid.

Dry eye is common in HED and results from a combination of mucus abnormalities from abnormal conjunctival mucinous glands, aqueous tear deficiency from abnormalities in the lacrimal gland, and lipid deficiency due to the partial or total absence of the meibomian glands.

In fact, in HED, defects in the meibomian glands are present in 95% of cases, with total or partial absence of the glands [34,35]. Therefore, HED patients are mainly affected by evaporative dry eye with rapid tear film break-up time (TBUT). Some authors also report a reduction of aqueous secretions of the lacrimal glands, evaluated by the Schirmer test and the tear lysozyme measurements [36,37]. Patients with combined lipid and aqueous tear abnormalities are more likely to have severe dry eye disease [38]. Most studies do not report objective ophthalmologic assessments of the ocular surface. However, a recent study evaluated a panel of ocular tests to diagnose and characterize dry eye in 34 patients with X-linked HED (8 infants aged 0–35 months, 12 male children aged 6–13 years compared to 6 healthy subjects aged 7–13 and 14 male adults aged 18–58 compared to 6 healthy subjects aged 23–47 years). Imaging the meibomian glands with meibography had 100% sensitivity and specificity for identifying XLHED. Meibography is the most reliable ophthalmic examination to establish a clinical diagnosis in individuals with suspected HED, even before genetic test results are available. Infrared thermography, a completely non-invasive procedure, revealed a typical pattern for male subjects with XLHED, but was less sensitive (86% for adults and 67% for children) than meibography or a combination of established routine tests. In adults, the ocular surface disease index and a non-invasive measurement of TBUT were the best single routine tests (sensitivity of 86% and 71%, respectively) to diagnose dry eye, whereas increased tear osmolarity appeared as a rather unspecific ophthalmic symptom. In children, non-invasive measurement of TBUT was the most convincing routine test (sensitivity of 91%) to detect tear film abnormalities. Tear film tests and ocular surface staining are very helpful for estimating the severity of ocular surface disease in individuals with known XLHED [39].

### 3.2. Anterior Segment and Eyelids

ED patients have circulating and mucosa-deposited anti-basement membrane zone autoantibodies, which may cause cicatrizing conjunctivitis unresponsive to topical therapy and lead to vision loss [40].

Corneal changes in HED are caused by the combined effect of the underlying ectodermal dysplasia [41], tear deficiency and recurrent ocular infection [15]. Consequent corneal opacities are present in approximately 19% of cases [42]. The most complete report of corneal changes in EDA was produced by Wilson et al., who reported a 13-year-old girl with EDA. This patient had reduced lacrimal secretion, diffuse punctate fluorescein staining of the cornea, and a superficial pannus of fine blood vessels in the corneal periphery with opacification. The corneal vascularization and pannus were progressive, requiring a superficial keratectomy. Histological examination of the corneal tissue showed epithelial acanthosis and dyskeratosis without keratinization, as well as replacement of the Bowman’s membrane with an inflammatory pannus of fibrovascular tissue [32].

A single clinical report described a male with clinical manifestations of HED and keratoconus, a progressive eye disease that gradually causes thinning of the cornea [36].

A case report by Zhang et al. described a 14-year-old male with HED with lower lid ectropion. The patient underwent surgical correction, and the lid remained in the normal position during the 10-month follow-up [43]. A more recent report by Chen et al. described successful correction of ectropion in 3 unrelated patients with ED through full-thickness free skin flap grafting with no ectropion recurrence noted after a 1-year follow-up period [44].

### 3.3. Lacrimal Drainage System

Patients with HED may develop anomalies of the lacrimal drainage system, such as the absence of lachrymal puncta, stenosis, or complete atresia of the nasolacrimal duct (NLD) [4,45]. Both primary-acquired, because of a congenital malformation, or secondary-acquired NLD obstruction, may develop in HED patients. The latter may be caused by atrophic rhinitis, a common condition in HED [46]. Beckerman reported a 16-year-old girl with EDA and bilateral epiphora due to complete NLD obstruction requiring a conjunctivo-dacryocystorhinostomy [41]. Liakos proposed that lacrimal defects were probably underrecognized as compromised lacrimal drainage was balanced by reduced tear production from lacrimal gland defects in EDA [47]. A single case report described a 12-year-old boy with dry eye and lacrimal sac mucocele, causing inverse ptosis in the lower eyelid and telecanthus (increased distance between the medial canthi), which was treated with dacryocystectomy [48].

### 3.4. Other Ocular Manifestations

A single case report described a 6-year-old African American male with HED and severe bilateral panuveitis with optic disc edema, peripheral retinal vasculitis, retinitis and macular epiretinal membrane. With all the other causes of panuveitis ruled out, the authors suggest that the abnormal development of tissue of ectodermal origin may predispose to inflammation and panuveitis [49]. A case of a child with HED, confirmed by molecular diagnosis, who presented with infantile bilateral glaucoma has been reported [50]. In another report, a 9-year-old child with HED presented with bilateral infantile glaucoma previously treated with trabeculectomy in both eyes at the age of 8 months. The child was treated with successful implantation of glaucoma with an Ahmed glaucoma valve [45]. Less commonly reported ocular manifestations include bilateral or unilateral congenital cataracts [51] and chorioretinal atrophy [4].

### 3.5. Molecular Basis of Ocular Phenotype in HED

**Figure 1:**
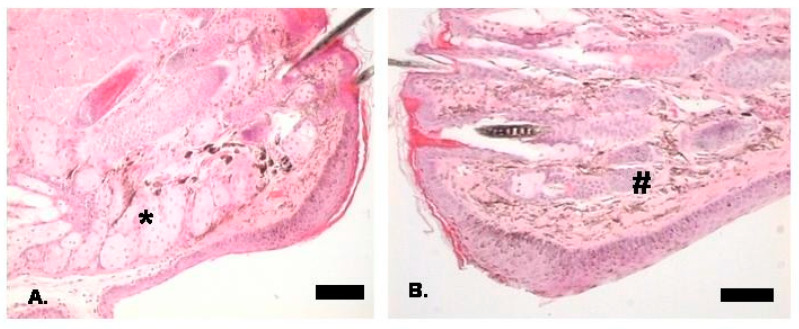
Histological appearances of the eyelid. (**A**)**.** Normal 129/Sv male mouse eyelid with * showing meibomian glands (H&E). Scale bar = 571 μm. (**B**). *Tabby* mouse showing complete absence of the meibomian glands with # indicating normal anatomical site of meibomian glands (H&E). Scale bar = 233 μm.

Understanding the molecular basis and dysregulated signalling pathways underlying the ocular phenotype in HED has come from studies using the XLHED *Tabby* (*Eda -/-*) mouse [20]. The ectodermal phenotype in the Tabby mouse reflects the impact of mutations in the human EDA1 gene. *Tabby* mice also exhibit absent meibomian glands (see Figure 1), dry eye features, ocular surface inflammation and corneal pathology [51,52]. Ocular surface inflammation in the *Tabby* mouse was dependent on housing conditions, with animals kept in conventional housing showing an increased susceptibility to inflammation compared to animals kept in pathogen-free barrier conditions [52]. There are also abnormalities in lacrimal gland development in the *Tabby* mouse, and *Eda* signalling plays a role in the development and function of the lacrimal gland [53]. *Eda* signalling has also been implicated in a cornea-lacrimal feedback loop in health and disease, which controls tear production [53]. Ectopic *Eda* expression in the *Tabby* mouse can rescue ocular surface disease even when the meibomian glands are absent [52]. The murine corneal epithelium and conjunctiva express the *Eda* receptor (EDAR) [54], which is mutated in human HEDs. *Eda* is highly expressed in murine meibomian glands and is detected in human tears [54]. The application of exogenous *Eda* improved the ocular surface, corneal cell proliferation and corneal wound healing in the *Tabby* mouse [54]. Exogenous *Eda* rescued epidermal growth factor (EGFR) signalling in the *Tabby* mouse. Reduced EGFR expression was previously reported in the *Tabby* mouse and human HED [55]. There is also evidence *Eda* upregulates the expression of tight junctional proteins (ZO-1 and claudin-1) via the sonic hedgehog signalling pathway to maintain corneal barrier dysfunction [56]. Understanding the molecular pathways disrupted in murine models of ED will help support the development of novel therapeutic strategies for the ocular phenotype of HED and potentially other corneal conditions [52,53,54,56,57].

## 4. Management of Ocular Manifestations

The management of EDs requires a multidisciplinary approach. Treatments for the ocular manifestations of HED are mainly supportive as they do not address the underlying molecular basis of the condition. Management of ocular surface disease requires specialized ophthalmological advice and supervision and includes: (1) regular, and sometimes intensive, preservative-free lubricants, which can be combined with puncta occlusion depending on the patency of the nasolacrimal system; (2) prevention of infections by reducing the staphylococcal load on the lids and ocular surface with lid hygiene, prophylactic topical antibiotics and staphylococcal decolonization strategies; (3) control of ocular surface inflammation with topical corticosteroids and immunosuppressants; (4) serum eyedrops, and given the underlying molecular genetic defect, allogenic serum is theoretically preferable; (5) surgical management of lash and lid disorders; and (6) careful and supervised use of specialized bandages and scleral contact lenses.

Moshirfar et al. recently published an article discussing higher rates of pre-, intra-, and post-operative complications of corneal refractive surgery in patients with ED. They also shared some recommendations for a more optimal approach to surgery and emphasized the importance of discussing those potentially increased risks with their patients [58].

Although the main ocular characteristics described are lack or reduction in eyelashes and meibomian glands, there are a wide range of underlying ocular signs which, if not attended to, would affect the patient’s vision and quality of life. Therefore, ocular investigation is mandatory in all patients with HED. Lastly, a comprehensive clinical and molecular diagnosis should be performed in patients with XLHED, considering that new therapies might be available in the upcoming years, rescuing the phenotype and its ocular manifestations.

## Data Availability

You may refer to the corresponding authors for any query.
